# Sleep duration and quality in permanent night work: an observational field study

**DOI:** 10.1007/s00420-024-02080-0

**Published:** 2024-06-21

**Authors:** Kirsten Nabe-Nielsen, Ann Dyreborg Larsen, Anne Emily Saunte Fiehn Arup, Mette Sallerup, Vivi Schlünssen, Åse Marie Hansen, Anne Helene Garde

**Affiliations:** 1 The National Research Centre for the Working Environment, Lersø Parkallé 105, 2100 Copenhagen, Denmark; 2 Department of Public Health, Section of Social Medicine, University of Copenhagen, Centre for Health and Society, Øster Farimagsgade 5, 1014 Copenhagen, Denmark; 3 Department of Public Health, Danish Ramazzini Centre, Research Unit for Environment, Occupation and Health, Bartholins Allé 2, 8000 Aarhus C, Denmark

**Keywords:** Consecutive night shifts, Chronotype, Industrial worker, Irregular working hours, Police, Shift work

## Abstract

**Purpose:**

Permanent night workers conceivably show better adaptation of circadian rhythms to night work than shift workers and therefore better possibilities of obtaining sufficient sleep of good quality after night shifts. We investigated the effect of night shifts including number of consecutive shifts on sleep among self-selected permanent night workers, and studied if the effect of night shifts differed between morning and evening types and compared with 3-shift workers.

**Methods:**

The study population included 90 permanent night workers followed for 14 days (warehouse workers, 1228 observation days, 80% males). For comparison, we included 70 3-shift workers followed for 26 days (police officers, 1774 observation days, 100% men). Total sleep time (TST), primary sleep duration (PSD), and sleep efficiency were assessed by actigraphy. Measures of sleep quality and diurnal type were self-reported.

**Results:**

Among permanent night workers, TST, PSD, difficulties falling asleep, disturbed sleep, and the number of awakenings decreased after night shifts compared with days without night work. Sleep efficiency, difficulties awakening, and non-refreshing sleep increased. More consecutive night shifts were associated with shorter TST and PSD. Sleep outcomes did not differ by diurnal type. Permanent night workers had fewer awakenings after night shifts than 3-shift workers, but no other differences were observed.

**Conclusion:**

This study does not provide evidence that supports recommendation of permanent night work to reduce adverse effects of night shifts on sleep. A limited number of consecutive night shifts is recommended to reduce accumulation of sleep debt.

## Introduction

In Denmark, 7.5% of the working population work night shifts as part of their primary occupation (The Danish Working Environment Authority 2018). Night work results in disturbed sleep, poor sleep quality, and short sleep (Boivin and Boudreau 2014; Costa 2015; Crowther et al. 2022; Sallinen and Kecklund 2010; Åkerstedt 2003) with potential long-term negative consequences for health (Kecklund and Axelsson 2016). Thus, to protect employee health, it is crucial to optimize the scheduling of night shifts to minimize the effect on sleep. Literature reviews conclude that a slowly backward-rotating work schedule implies more sleep impairments than a fast forward-rotating work schedule (Bambra et al. 2008; Driscoll et al. 2007; Sallinen and Kecklund 2010). Additionally, some studies find that sleepiness is most pronounced on the first night shift and that sleep duration and alertness increase with more consecutive night shifts (Garde et al. 2020a). Other data do not support this finding (Garde et al. 2020b; Jensen et al. 2022b), and a recent Cochrane review questions the presence of any evidence of an effect of specific features of a shift work schedule on sleep (Hulsegge et al. 2023).

Disturbances of circadian rhythms and internal desynchronization occurring when employees shift between day and night orientation are suggested as mechanisms through which night work influences health (Jensen et al. 2015; Kecklund and Axelsson 2016). Permanent night work, i.e. a shift schedule containing solely night shifts and days off, allows (in principle) for better adaptation to daytime sleep and nighttime activity, because permanent night workers do not have to change their diurnal rhythms between night shifts in order to work day shifts. Along this line of reasoning, it could be hypothesized that permanent night work for a smaller (selected) group may be a better option than having more employees working night shifts in combination with other shifts. For example, sleep after night shifts has been shown to be longer in permanent night shift systems (6.65 h) than in rotating shift work (5.85 h) (Pilcher et al. 2000). Furthermore, in one study, permanent night workers had fewer insomnia symptoms after working a night shift compared with 3-shift workers covering day, evening and night shifts, while on days off, the latter group showed fewer insomnia symptoms compared with permanent night workers (Flo et al. 2013). Additionally, some people prefer night shifts (Barton et al. 1993; Nabe-Nielsen et al. 2010), and being an evening type and having higher sleep flexibility are associated with higher tolerance to shift work (Ritonja et al. 2019; Saksvik et al. 2011). Thus, individual factors could modify the effect of night work on sleep. Yet, even permanent night workers are likely to being awake during the day on days off due to family and social activities, and it has been shown that only a very small minority (0.3%) of permanent night workers show “complete” adjustment of their endogenous melatonin rhythm to night work (Folkard 2008; Knauth and Rutenfranz 1977). Thus, based on the current literature, it is still unclear if night shifts should preferably be organized as permanent night work or as part of a rotating 2- or 3-shift schedule in order to minimize effects on sleep and thereby potential consequent effects on health and safety.

Therefore, the first aim of the current study was to investigate, how night shifts affected sleep in a group of permanent night workers. Second, we analyzed the effect of the number of consecutive night shifts in order to take the relative timing of each shift into account. Third, we investigated if an individual characteristic, in terms of diurnal type, modified the effect of night shifts on sleep. Fourth, we investigated if the effect of night shifts on sleep differed between permanent night workers and 3-shift workers. We hypothesized that working night shifts affected permanent night workers’ sleep, but to a smaller extent than among 3-shift workers. This expected difference could be due to a combination of selection mechanisms and adaptation to night work. We also expected that more consecutive night shifts resulted in an increasingly longer and better daytime sleep due to adaptation to working at night, and that evening types had longer sleep of better quality after night shifts than morning types.

## Methods

### Data sources and participants

The present study includes *permanent night workers* from the observational field study “*Accidents and health among permanent night workers”* (data collection period January 2019-September 2020; one individual collected data during a regular COVID-19 related lock-down) and *3-shift workers* from the quasi-experimental crossover study “*In the Middle of the Night”* (data collection period: January 2013-November 2013). The National Committee on Health Research Ethics in Denmark approved the studies (protocol numbers H-18046118 and H-4–2012-155). All participants signed a declaration of consent. The study population and approach to the scheduling of night shifts differed between the two studies. Otherwise, the data collection followed the same protocol. Paper-versions of questionnaires and sleep diaries were distributed to the participants, who filled them in independently at the workplace or at home without the presence of a researcher.

### Design and recruitment

From the study *“Accidents and health among permanent night workers”* we included 90 participants with complete information on sex and age (based on experiences from previous field studies, we aimed at including at least 20 participant to obtain sufficient statistical power). Participants were requested to fill in sleep diaries after their primary sleep for 14 consecutive days. During this period, the participants worked night shifts (757 observation days) and had recovery days (471 observation days). Recovery days mainly included days off. The most prevalent work schedules were 4 + 3 schedule (4 consecutive nights followed by 3 consecutive recovery days), a 4 + 2 or a 5 + 2 schedule.

The permanent night workers were recruited from 4 companies within wholesale and retail trade, which signed up for the project. The participants were mainly involved in warehouse work, e.g., truck driving and industrial production. Employees were informed about the project in connection with a health examination at the workplace, performed by the private consultancy bureau, CRECEA, or at information meetings with researchers at their workplace. Hereafter, employees could individually sign up for the project. Participants were instructed to initiate data collection when waking up from their primary sleep after their first night shift in a set of night shifts.

The study *“In the Middle of the Night”* was initiated with the purpose of investigating the effect of the number of consecutive night shifts on sleep (Garde et al. 2020b), hormones (Jensen et al. 2016), and heart-rate variability (Jensen et al. 2022a). Male police officers were recruited from the 5 police districts at Zeeland, Denmark. Information meetings were held for the leaders, the people responsible for personnel-on-duty planning, and employee representatives. All districts were also offered an information meeting for potential participants, and an e-mail was sent to all potential participants in all districts with an invitation to participate in the study.

As part of their normal work, all participants worked shifts including nights, and they worked either in a call center or on patrol. According to the protocol, each participant should complete three different work schedules decided by the researchers during the study period. Each work schedule was applied only once for each participant, and all three should be completed within a 3-month period. The three work schedules consisted of a 2 + 2 schedule (2 night shifts followed by two recovery days), a 4 + 4, and a 7 + 7 schedule. Participants were followed, i.e. filled in sleep diaries, for a maximum of 26 days. Recovery days could be day shifts or days off. The participants did not work any night shifts in the seven days leading up to the start of the three experimental work schedules. In total, 70 male police officers worked at least one of the three schedules and provided information about age (935 observed night shifts and 839 observed recovery days). The design and recruitment is described in more detail elsewhere (Jensen et al. 2016).

### Data collection

#### Background factors

In a background questionnaire, participants were asked about sex, birth year, tenure, years of night shift experience, self-rated health, diurnal type, and their preferred shift type (Table 1). We also asked about perceived advantages of working night shifts.

**Table 1:** Description of the study populations. For all variables included only for descriptive purposes, participants with missing values were excluded analyses by analyses. Participants with missing values on sex and age were excluded from the study population

	Permanent night workers (n=90)				3-shift workers (n=70)			
	N	%	Mean	SD	N	%	Mean	SD
Sex								
Female	18	20%			0	0		
Male	72	80%			70	100%		
Age (years)			48.6	11.7			38.4	9.6
Tenure (years)			12.8	12.0			10.8	9.6
Night work experience (years)			13.6	10.5			11.2	8.1
Self-rated health								
Excellent	9	10%			22	32%		
Quite good	32	36%			32	46%		
Good/less good/bad	49	54%			15	22%		
Diurnal type								
Morning/more morning than evening	32	37%			19	27%		
Evening/more evening than morning	55	63%			51	73%		
Prefers day work^1^	28	41%			25	36%		
Prefers evening work^1^	4	6%			38	54%		
Prefers night work^1^	72	85%			27	39%		
Experience night shifts as strenuous								
Score 1–2 (not at all strenuous)	38	44%			14	20%		
Score 3–5	41	48%			50	71%		
Score 6–7 (very strenuous)	7	8%			6	9%		
Total and mean number of observed days	1228	100%	13.6	1.0	1774	100%	25.3	2.4
Distribution of “exposure conditions”								
Night shifts	757	62%			935	53%		
Recovery days (all)^2^	471	38%			839	47%		
Shift number^3^								
1	140	30%			134	23%		
2	111	23%			134	23%		
3	96	20%			95	16%		
4	79	17%			88	15%		
5	39	8%			47	8%		
6–8	9	2%			88	15%		
Total	474	100%			586	100%		

^1^Often/always (vs. never/seldom/sometimes, not shown)

^2^Of the recovery days, 7 were followed by a morning/day shift starting before 12:00 noon among permanent night workers, and this was the case for 224 of the recovery days among 3-shift workers; 64 and 221 shifts could not be classified as we lacked information about working hours following the shift

^3^Only night shifts after an observed recovery day are shown (including “last shift in a set of night shifts”)

#### Self-reported sleep

Participants were instructed to fill in sleep diaries immediately after their primary sleep, i.e. their main sleep period on that day. Participants also responded to questions about (I) difficulties falling asleep (*Not at all* to *Very much*), (II) disturbed sleep (*Not at all* to *A lot*); (III) number of awakenings (*0 times*–*4 or more times*), (IV) difficulties awakening (*Very easy* to *Very difficult*), (V) refreshing sleep (*Very refreshing* to *Not at all refreshing*), and (VI) how the sleep was (i.e. sleep quality) (*Very good* to *Very poor*). Items I–VI were derived from the Karolinska Sleep Questionnaire (Kecklund and Åkerstedt 1997; Åkerstedt et al. 2002). All items had five response options and were coded from 1 to 5 with higher scores representing poorer sleep. Due to the ordinal response options, these variables were treated as continuous in the statistical analyses, instead of—for instance—a dichotomization, in order to maintain as much information as possible.

#### Objective measures of sleep

Actigraphs (ActiGraph wGT3X-BT from ActiGraph Florida, USA) were worn on the wrist of the non-dominant hand. Participants were asked to wear the actigraph during the whole 24 h day, but most importantly during sleep episodes. Data were collected with a sampling rate of 30 Hz, and 1 min epochs were used to score sleep. Data were analyzed with ActiGraph Sleep Analysis (ActiGraph Florida, USA). We extracted total sleep time per day including naps (TST), primary sleep duration (PSD), and sleep efficiency (i.e. percentage of time asleep relative to time in bed for primary sleep). Information about when participants got into and out of bed for all sleep episodes was obtained from sleep diaries, and this information was used as input when extracting data from the actigraphs.

#### Work schedules

Information about work schedules was obtained from the sleep diaries. Participants indicated, if they had been at work during the preceding 24 h including the starting and ending times of the shift. Night shifts were defined as shifts covering at least 3 h between 23:00 and 06:00.

Among *permanent night workers*, the most frequent night shifts were 21:25–6:30 (200 observations), 18:00–3:05 (68 observations), and 23:00–7:00 (60 observations). The remaining night shifts started at different time points between 16:45 and 00:00 and ended at different time points between 02:00 and 9:00. The mean night shift duration was 8:37 hh:mm. Among *3-shift working police officers*, the most frequent night shift was 23:00–7:00 (487 observations), 22:45–6:45 (91 observations), or 22:00–6:00 (87 observations). The remaining night shifts started at different time points between 17:00 and 2:30 and ended at different time points between 2:00 and 13:00. The mean night shift duration was 8:07 hh:mm.

Shift number was coded for permanent night workers. To ensure valid information about number of consecutive night shifts, we only included observations starting from the first night shift after an observed recovery day occurring during the study period. For the 3-shift workers, each experimental work schedule was preceded by 7 days without night work. Therefore the number of consecutive night shifts was counted from the first observed night shift in each schedule. In the current study, these latter data are only reported for descriptive purposes, as the effect of consecutive night shifts in this group has been reported elsewhere (Garde et al. 2020b).

The comparison of sleep outcomes between permanent night workers and 3-shift workers could be hampered by systematic differences in the “recovery day condition” in the two groups. Particularly, sleep may be curtailed by morning/day shifts, and the group of 3-shift workers were more likely to have a scheduled day shift during their recovery period than the permanent night workers. Therefore, we subdivided the observed recovery days into recovery days where nighttime sleep was followed by a day off/a day shift starting not earlier than 12:00 and recovery days where nighttime sleep was followed by a morning/day shift starting before 12:00.

### Statistical analysis

Descriptive data are presented in Table 1. The sample sizes for the descriptive analyses vary due to missing values for some of the variables. For permanent night workers, mean and standard deviation (SD) of sleep after night shifts and on recovery days are presented in Table 2.

**Table 2:** Comparison of sleep after night shifts and on recovery days in 90 permanent night workers

	After night shifts		On recovery days		Difference between night shifts and recovery days		
	Mean	(SD)	Mean	(SD)	Estimate	Lower 99% CI	Upper 99% CI
Total sleep duration (hh:mm)^1^	05:39	01:20	07:24	01:50	**−01:48**	**−02:02**	**−01:35**
Primary sleep duration (hh:mm)^2^	05:16	01:20	06:55	01:42	**−01:40**	**−01:52**	**−01:27**
Sleep efficiency (%)^2^	88.6	9.1	87.9	7.6	**1.11**	**0.03**	**2.20**
Difficulty falling asleep	1.6	0.8	1.9	1.1	**−0.29**	**−0.42**	**−0.16**
Difficulty of awakening	2.4	0.9	2.3	0.8	**0.12**	**0.01**	**0.23**
Non-refreshing sleep	2.9	0.9	2.7	0.9	**0.27**	**0.15**	**0.39**
Disturbed sleep	1.8	0.9	1.9	1.0	**−**0.07	**−**0.18	0.05
No. of awakenings	2.2	1.1	2.4	1.2	**−0.17**	**−0.32**	**−0.02**
Poor sleep quality	2.1	0.8	2.2	0.9	0.01	**−**0.10	0.11

^1^All sleep episodes

^2^Data from primary sleep or longest nap if no primary sleep is indicated.

Descriptive data are presented as means and standard deviations (SD). Estimates represent the estimated difference in means with their 99% Confidence Intervals (CI) adjusted for sex and age. N=1228 observations (1–17 missing observations per analysis; excluded analysis by analysis).

For the analyses of the association between night shifts and sleep, we performed repeated measures ANOVA using the PROC MIXED procedure with a random intercept for each individual to account for within-subject variation. In the *first* set of analyses, we analyzed how sleep after night shifts differed from sleep on recovery days by including data from *permanent night workers* and across all measurement days (Table 2).

In the *second* set of analyses, we analyzed the association between number of consecutive night shifts and sleep (Table 3). Data from the *last* night shift in a set of consecutive night shifts were analyzed separately, because we previously demonstrated that these sleep episodes are shortened as a preparation for returning to day time orientation (Garde et al. 2020b). Furthermore, due to very few observations (< 10) from night-shift number 5, 6, and 7, the analysis was restricted to night-shift number 1–4 (Table 3). In the *third* set of analyses, we compared sleep for morning types and evening types on recovery days and after night shifts, excluding 3 participants with missing information on diurnal type (Table 4). All analyses were adjusted for sex and age.

**Table 3:** Changes in sleep with more consecutive night shifts (n=327 observations) and differences in sleep between last night shift vs. other night shifts (n=426 observations)

	Effect of number of consecutive night shifts			Effect of “last night shift” vs. all other night shifts		
	Beta	Lower 99% CI	Upper 99 % CI	Beta	Lower 99% CI	Upper 99 % CI
Total sleep duration (hh:mm)^1^	**−00:13**	**−00:22**	**−00:04**	**−00:44**	**−01:04**	**−00:25**
Primary sleep duration (hh:mm)^2^	**−00:09**	**−00:17**	**−00:01**	**−00:34**	**−00:52**	**−00:15**
Sleep efficiency^2^	**−**0.03	**−**0.90	0.84	**−**1.59	**−**3.45	0.27
Difficulty falling asleep	**−**0.04	**−**0.14	0.06	0.17	**−**0.04	0.39
Difficulty of awakening	0.03	**−**0.06	0.12	**−**0.04	**−**0.23	0.16
Non-refreshing sleep	**−**0.02	**−**0.12	0.08	0.17	**−**0.04	0.39
Disturbed sleep	**−**0.05	**−**0.15	0.05	**−**0.18	**−**0.39	0.02
No. of awakenings	**−**0.11	**−**0.24	0.01	**−0.31**	**−0.59**	**−0.04**
Poor sleep quality	**−**0.04	**−**0.13	0.05	**−**0.04	**−**0.24	0.15

Significant estimates are in bold

^1^All sleep episodes

^2^Data from primary sleep or longest nap if no primary sleep is indicated

Only data from night shift 1–4 are included. Effects are presented as beta-values (i.e. the slope) of the change in sleep with one additional consecutive night shift with their 99%. Confidence Intervals (CI) adjusted for sex and age. 1–6 missing observations per analysis (excluded analysis by analysis)

**Table 4:** Comparison of sleep outcomes on recovery days (n=471 observations) and after night shifts (n=757 observations) among permanent night workers describing themselves as morning types and evening types (reference)

	Morning types compared with evening types (reference) on recovery days			Morning types compared with evening types (reference) after night shifts			Interaction
	Estimate	Lower 99% CI	Upper 99% CI	Estimate	Lower 99% CI	Upper 99% CI	p-value^3^
Total sleep duration (hh:mm)^1^	**−**00:17	**−**00:55	00:21	**−**00:11	**−**00:36	00:15	0.423
Primary sleep duration (hh:mm)^2^	**−**00:29	**−**01:06	00:09	**−**00:15	**−**00:44	00:14	0.180
Sleep efficiency^2^	**−**1.39	**−**4.54	1.77	**−**1.12	**−**4.75	2.52	0.735
Difficulty falling asleep	0.35	**−**0.08	0.77	0.09	**−**0.19	0.38	**0.005**
Difficulty of awakening	**−**0.02	**−**0.36	0.33	**−**0.02	**−**0.34	0.30	0.689
Non-refreshing sleep	0.11	**−**0.28	0.51	0.17	**−**0.15	0.50	0.605
Disturbed sleep	0.16	**−**0.24	0.56	0.09	**−**0.24	0.41	0.358
No. of awakenings	0.46	**−**0.06	0.98	0.27	**−**0.15	0.69	0.203
Poor sleep quality	0.26	**−**0.08	0.61	0.18	**−**0.11	0.47	0.174

Significant estimates are in bold. Interaction analyses test interaction between diurnal type (morning type vs. evening type) and the night shift vs. recovery day condition

^1^ All sleep episodes

^2^ Data from primary sleep or longest nap if no primary sleep is indicated

^3^ p for interaction

Estimates represent the estimated difference in means with their 99%

Confidence Intervals (CI) adjusted for sex and age. 14–34 missing observations per analysis (excluded analysis by analysis)

In the *fourth* set of analyses, we combined data from *permanent night workers* and *3-shift workers* and analyzed if the effect of night shifts on sleep differed between these two groups (Table 5). These analyses were restricted to males, and we adjusted for age. Only recovery days, where sleep was not followed by a shift starting before 12:00, were included.

**Table 5:** Comparison of sleep outcomes on recovery days (n=717 observations) and after night shifts (n=1549 observations) when comparing 72 permanent night workers and 70 3-shift workers (reference)

	Permanent night workers compared with 3-shift workers (reference) on recovery days			Permanent night workers compared with 3-shift workers (reference) after night shifts			Interaction
	Estimate	Lower 99% CI	Upper 99% CI	Estimate	Lower 99% CI	Upper 99% CI	p-value^3^
Total sleep duration (hh:mm)^1^	00:16	**−**00:13	00:44	00:08	**−**00:13	00:28	0.127
Primary sleep duration (hh:mm)^2^	**−**00:04	**−**00:33	00:24	00:22	**−**00.00	00:44	0.018
Sleep efficiency^2^	0.31	**−**2.65	3.27	1.33	**−**1.37	4.03	0.701
Difficulty falling asleep	0.10	**−**0.25	0.46	0.07	**−**0.13	0.28	0.829
Difficulty of awakening	**−**0.15	**−**0.45	0.15	0.02	**−**0.23	0.27	0.019
Non-refreshing sleep	**−**0.01	**−**0.33	0.31	**−**0.20	**−**0.46	0.07	0.025
Disturbed sleep	**−**0.09	**−**0.40	0.23	**−**0.15	**−**0.39	0.09	0.597
No. of awakenings	**−**0.33	**−**0.77	0.11	**−0.39**	**−0.74**	**−0.05**	0.684
Poor sleep quality	0.05	**−**0.01	0.01	**−**0.04	**−**0.27	0.19	0.130

Significant estimates are in bold. Interaction analyses test interaction between group (permanent night worker vs. 3-shift worker) and the night shift vs. recovery day condition. The latter only includes sleep not followed by a day shift starting before 12:00 noon

^1^All sleep episodes

^2^Data from primary sleep or longest nap if no primary sleep is indicated

^3^p for interaction. Estimates represent the estimated difference in means with their 99%

Confidence Intervals (CI) adjusted for age. 3–53 missing observations per analysis (excluded analysis by analysis)

For all analyses of differential effects between groups, formal tests of interactions were performed by introducing a product term of the two main variables to a statistical model (i.e. *“night shifts* + *diurnal type* + *night shifts×diurnal type”* and *“night shifts* + *group* + *night shifts×group”*). The significance levels of the interaction terms are shown in Tables 4 and 5.

Results are presented as the estimated differences in the means or as beta-values (specified for each Tables 2–5) with their 99% confidence intervals (CI). An alpha level of 0.01 (i.e. p < 0.01) was used to take multiple statistical testing into account, and significant differences at a 1%-level are in bold in Tables 2–5. All statistical analyses were performed using the statistical software SAS for Windows 9.4 (TS level 1M3, SAS Institute, Cary, NC).

## Results

Permanent night workers consisted primarily of men (80%), and the 3-shift workers consisted solely of men (Table 1). The former group was 10 years older on average, had a longer tenure in their current workplace, and had more years of night-work experience. Self-rated health was poorer among permanent night workers. The percentage of morning types was slightly higher among permanent night workers, yet 85% of permanent night workers preferred to have night shifts often/always rather than never/seldom/sometimes, while this was the case for 39% of the 3-shift workers. Permanent night workers also found night work substantially less strenuous, and the most prevailing advantages of working night shifts in this group were: “At night, there is a better atmosphere at the workplace” (reported by 77%), “It is an economic advantage” (reported by 70%), “It fits well with my private life and my family life” (reported by 53%), and “I like to be off work during the daytime” (reported by 50%).

Among permanent night workers, we observed that TST was significantly reduced with 01:48 h and PSD was reduced with 01:40 h after night shifts compared with recovery days (Table 2). We observed a minor (but significant) increase in sleep efficiency of 1.11% points. We also found a decrease in difficulties falling asleep and number of awakenings as well as an increase in difficulties awakening and non-refreshing sleep.

TST and PSD decreased with increasing numbers of consecutive night shifts (00:13 hh:mm and 00:09 hh:mm per extra night shift, respectively) (Table 3). When comparing sleep after the last night shift in a row with all other night shifts, we found that TST and PSD were shorter and that fewer number of awakenings were reported.

We did not find any major differences in sleep when comparing morning types and evening types on recovery days and after night shifts. Yet, we found that the difference between morning types and evening types regarding their difficulties falling asleep after night shifts was smaller than expected when taking the differences between the groups on recovery days into account (Table 4, p = 0.005).

We did not find any significant differences in sleep, when comparing permanent night workers and 3-shift workers on recovery days and after night shifts, except for a lower number of awakenings after night shifts among permanent night workers. We found indications of a longer PSD among permanent night workers after night shifts, than among 3-shift workers, but both the point estimate and p for interaction were only borderline statistically significant at a 1%-level (Table 5).

## Discussion

### Main findings

Permanent night workers had shorter sleep after night shifts than on recovery days, and sleep efficiency increased along with corresponding self-reported measures, such as fewer difficulties falling asleep and fewer awakenings. Still, they had more difficulties waking up and reported more non-refreshing sleep after night shifts.

Our data did not support the hypothesis that sleep improved with more consecutive night shifts. Actually, TST and PSD decreased with an increasing number of consecutive night shifts among permanent night workers. We did not find substantial evidence of a stronger effect of night shifts on sleep among morning types compared with evening types. Furthermore, we did not find support for the hypothesis that permanent night workers adapted better to night shifts than 3-shift workers as sleep did not seem to differ significantly between the two groups—apart from the reporting of fewer awakenings after night shifts among permanent night workers.

### Comparison with previous findings

The debate about organization of night work and the pros and cons of permanent night work dates back to the nineties (Barton 1994; Dirkx 1993; Wilkinson 1992). One argument for permanent night work is that permanent night workers have the possibility of adapting to nighttime activity and daytime sleep (Folkard 2008), thereby reducing sleep debt after night shifts. Among permanent night workers in the current study, both TST and PSD were substantially curtailed after night work. Permanent night workers also had higher sleep efficiency, less difficulty falling asleep, and fewer awakenings after night shifts compared with recovery days, but sleep was also less refreshing and more difficult to wake up from. We interpret these findings as signs of a higher sleep pressure caused by sleep deprivation in combination with misalignment of sleep with the circadian rhythm.

In our study, permanent night workers did not demonstrate better sleep after night shifts than 3-shift workers. Therefore, when looking at sleep in isolation, our data indicate that permanent night workers do not cope better with night shifts than 3-shift workers.

In a comparison of nurses working either permanent night shifts or 3-shift work, the latter group reported poorer sleep after night shifts. Yet, when being on vacation or not working, permanent night workers reported being more tired and sleepy. Permanent night workers also had lower odds of night-shift insomnia and higher odds of rest-day insomnia than 3-shift workers (Flo et al. 2013). These results contradict our findings, as we did not find any substantial differences between permanent night workers and 3-shift workers. Another study among nurses reported that sleep duration after night shifts was similar among 3-shift workers and permanent nights workers, but the latter group had shorter sleep on days off (Garde et al. 2009). These findings are also partly opposite to ours, as we found no difference in TST and PSD on recovery days, which were mainly days off, a (non-significantly) longer PSD after night shifts, and no difference in TST after night shifts.

A German study of industrial workers showed that permanent night workers slept 5:41 hh:mm after night shifts and 8:13 hh:mm on work-free days (Casjens et al. 2022). Especially, sleep duration after night shifts seems comparable to our finding. Participants working nights as part of a rotating schedule with 8 h shifts, slept 5:03 hh:mm after night shifts and 7:17 hh:mm on work-free days. Thus, in the previous study, permanent night workers seemed to have the longest sleep duration. The German study also demonstrated poorer sleep quality among permanent night workers (Casjens et al. 2022), which we did not replicate.

We did not find that more consecutive night shifts resulted in longer or better sleep among permanent night workers. On the contrary, we observed that sleep duration decreased with more consecutive night shifts. Thus, our findings do not challenge existing recommendations of a maximum of 3 consecutive night shifts in order to minimize sleep debt, and health and safety risks (Garde et al. 2020a). These recommendations possibly contradict some shift workers’ preferences for working more consecutive night shifts to obtain longer periods of days off or because “the first night shift is the worst” (Nabe-Nielsen et al. 2016, 2010). A previous laboratory study found that response latency and lapse frequency decreased with more consecutive night shifts, which appears as an argument for longer spells of night work (Lamond et al. 2004). On the contrary, accidents seem to increase with more consecutive night shifts (Fischer et al. 2017).

Surprisingly, in our study, permanent night workers were not more prone to being evening types than the 3-shift workers. Permanent night workers may have chosen night work for other reasons than diurnal preferences, and the atmosphere at the workplace, economic incentives, and private/family life were indicated as advantages of night work. We did not find any substantial differences in sleep duration or sleep quality between evening and morning types, although our results suggests that morning types had more difficulties falling asleep than evening types on recovery days, whereas the difference between the groups diminished after night shifts. Interestingly, a previous study found that morningness was associated with higher tolerance to day shifts operationalized as insomnia symptoms and sleepiness/tiredness, whereas morningness was not associated with tolerance to evening or night shifts (Storemark et al. 2013).

Taken together, evidence consistently support that sleep is shorter after night work than on recovery days (i.e. primarily days off in our study). Yet, when comparing with other studies, the absolute level seems to vary across populations and settings. Our study indicates that perhaps permanent night workers’ PSD is longer and their sleep could be less interrupted after night shifts than for 3-shift workers, but this finding warrants further investigation. Sleep duration, sleep quality and fatigue, and perhaps also other indicators such as physical and psychological well-being across a whole shift cycle could be relevant outcomes.

### Strengths and limitations

The strength of our study lies in its inclusion of day-to-day data from 90 permanent night workers and 70 3-shift workers with a total 3002 observation days. Thus, we observed the same individuals under different conditions i.e. night shifts and recovery days. Inclusion of self-reported data on working hours and sleep collected via sleep diaries combined with objectively measured actigraphy data also strengthens the study. The data collection for the 2 study populations followed the same protocol. Yet, while the study among permanent night workers was observational, the work schedule among the 3-shift workers was manipulated during the study period.

The primary study population was a group of permanent night workers. This group of participants could be selected into night work because of a preference for working night shifts, which is supported by our descriptive data. Yet, diurnal type did not seem to be associated with a preference for permanent night work. While selection is typically a bias, the purpose of the present study was indeed to investigate the effect of night work on sleep in employees, who selected themselves into permanent night work in a real-life setting. Therefore, this self-selection is not considered a limitation in the current study. We included data from 3-shift working police officers as a comparison group. For this group of employees, working night shifts is part of the job, yet they also reported some advantages related to night work, i.e. variation in everyday life, having time off during the day, and economic advantages (Nabe-Nielsen et al. 2016). In the current analyses, they provide us with an estimate of the effect of night work on sleep in a healthy population, who is not expected to be selected into night work per se.

The two groups differ, e.g., in terms of occupational group, age, and preferences for night work. In addition, the permanent night workers worked their usual schedule, while the police officers’ work schedules were determined by the study protocol. Thus, while the 3-shift workers were acquainted with shift work, they were not necessarily familiar with the specific 2 + 2, 4 + 4 or 7 + 7 system, which could have influenced their sleep. Furthermore, we cannot rule out that unmeasured characteristics of the work schedules, could have influenced our findings. For example, the work performed by the participants differed, and in the present study, the effect of night work solely in terms of *time of the day*, would be difficult to isolate from the effect of the work performed (e.g., high physical, emotional or mental demands). Indeed, permanent night workers found night work less strenuous, which could both be due to adaptation to working at night and due to the actual tasks performed. Such differences should be taken into consideration when interpreting the results, as they may contribute to the effects of different shift systems on health. Importantly, however, our study contribute to knowledge about night work and health in a real-life setting in which exposures usually cluster.

Overall, we did not observe substantial differences in sleep duration and quality between permanent night workers and shift workers on recovery days or after night shifts. Additionally, while permanent night workers’ recovery day-sleep was seldom curtailed by a scheduled day shift, this was often the case among shift working police officers, and to strengthen the study, we excluded these observations from the comparison of the two groups. Interestingly, a previous study among oilrig workers reported that the group of night shift workers slept longer after night shifts (6.4 h) than after day shifts (5.8 h) (Bjorvatn et al. 2006). Thus, when evaluating the effect of night work on sleep and other acute outcomes in different occupational groups, contextual factors and “contrast conditions” are important to consider.

In the analyses comparing 3-shift workers and permanent night workers, only men were included. In general, we expect the biological mechanisms behind night work and sleep to be similar for men and women, but we cannot elucidate sex differences in the effects of different types of night shift schedules on sleep, and the group of women was too small to run separate analyses. This is, however an important question for future research, as a previous study suggests differential effect of night shift schedules on men and women when looking at metabolic outcomes (Bayon et al. 2022).

### Conclusion and implications

In alignment with the prevailing evidence in this field, we found that night shifts resulted in shorter sleep compared with recovery days among permanent night workers. Presumably due to sleep debt and misalignment of circadian rhythms, permanent night workers seemed to find it easier to fall asleep and maintain sleep after night shifts, but also seemed to experience insufficient recovery as they had difficulties wakening up and they felt less refreshed.

Additionally, we did not find support for the notion that more consecutive night shifts benefit permanent night workers, as sleep duration decreased with more night shifts. Likewise, self-reported diurnal type did not seem to have a major influence on the effect of night shifts on sleep.

Overall, the current study does not provide strong support for the notion that those, who choose permanent night work, have longer or better sleep than shift workers after night shifts. Therefore, our findings do not support the concentration of permanent night work in a smaller, selected group to prevent adverse effects of shift work on sleep and subsequent health and safety risks, and in the case of permanent night work, it is recommendable to limit the number of consecutive night shifts to reduce sleep debt.

Despite that our results on night shifts and sleep, per se, do not support an advice against permanent night work, permanent night work would lead to a higher lifetime accumulation of night shifts compared with 2- or 3-shift schedules, which should also be taken into consideration, when evaluating pros and cons of approaches to night shift scheduling. Our findings can contribute to the knowledge base for employers and occupational health professionals on the effect of permanent night work on sleep. This knowledge can guide scheduling of night shifts benefitting health for employees in various occupations.

## Data Availability

Data involves identifiable human data and only an anonymized version can be shared publicly. Access to data can also be obtained by researchers affiliated with the National Research Centre for the Working Environment (employed or guest researchers) while adhering to restrictive terms of use.
